# Case Report: A rare case of young adult progressive familial intrahepatic cholestasis-type 3 with a novel heterozygous pathogenic variant of *ABCB4*

**DOI:** 10.3389/fped.2022.1012825

**Published:** 2022-10-18

**Authors:** Hao Zhu, Shengnan Wang, Li Li, Wenqian Geng, Xiaoqiang Wan, Rui Hua, Dong Wang, Pujun Gao

**Affiliations:** ^1^ Department of Hepatology, The First Hospital of Jilin University, Changchun, China; ^2^ Department of Neurology, The First Hospital of Jilin University, Changchun, China; ^3^ Department of Interventional Therapy, The First Hospital of Jilin University, Changchun, China

**Keywords:** ABCB4, progressive familial intrahepatic cholestasis type 3, novel mutation, multidrug-resistant protein 3, c.1429C>A

## Abstract

Progressive familial intrahepatic cholestasis type 3 (PFIC-3) is a rare autosomal recessive disorder with poor prognosis. It is caused by pathogenic variants of the ATP binding cassette subfamily B member 4 (*ABCB4*) gene and usually progresses from chronic cholestasis with or without jaundice to portal hypertension and end-stage liver disease within the first to second decade of life. Few reported PFIC-3 patients presented with atypical clinical symptoms, therefore, often misdiagnosed if without family history. Herein, we report a 16-year-old male who was admitted to our hospital due to acute episodes of jaundice and intense pruritus, subsequently progressed to end-stage liver disease. Laboratory examinations showed no evidence of liver injury caused by viral, autoimmune, drug or liver tumors. Ursodeoxycholic acid and dexamethasone did not relieve his symptoms and he underwent liver transplantation successfully. Targeted next-generation sequencing identified that the patient was a compound heterozygote for two missense mutations (c.959C > T/c.1429C > A) in the *ABCB4* gene. The mutation c.1429C > A (p.Q477K) is a novel heterozygous mutation. We constructed a three-dimensional model of this novel pathogenic variant using the SWISS MODEL program and found that the patient's *ABCB4* protein is an ATP hydrolysis deficient mutant. The postoperative pathological diagnosis showed intrahepatic cholestasis with progression to cirrhosis. Negative liver tissue immunohistochemistry of MDR3 was found in the explanted liver. The patient was diagnosed with PFIC-3, and his symptoms improved dramatically with liver transplantation. In conclusion, for young patients with acute cholestasis, pruritus, jaundice, growth retardation, and enlargement of the liver and spleen, the possibility of inherited metabolic liver diseases should be considered, detailed medical and family history should be collected, and metabolic screening tests as well as gene tests are necessary for correct diagnosis. Increasing the coverage of PFIC3 is meaningful and thus can improve the current understanding of this disease.

## Introduction

Progressive familial intrahepatic cholestasis (PFIC) is a group of rare diseases that occurs in the neonatal or infancy period. Based on clinical presentations, laboratory findings, liver histology, and genetic defect, they can be broadly divided into six types of PFIC (1). Among them, the classical PFIC type 1 (PFIC-1), PFIC type 2 (PFIC-2), and PFIC type 3 (PFIC-3) are more common (2). The main clinical manifestations of PFIC include cholestasis, pruritus, jaundice, growth retardation, and often enlargement of the liver and spleen. PFIC presents a progressive course and will progress to cirrhosis and liver failure within the first 10 years of life whereas few patients may have a slow progression. PFIC-1 and PFIC-2 are relatively more common with an overall estimated incidence of 1 per 50,000 to 1 per 100,000 births (2), while PFIC-3 are especially rare, and the overall estimated incidence is 1 per 500,000 births (3). PFIC-1 usually debuts within the first year of life, and extrahepatic disease manifestations (diarrhoea, pancreatitis, pneumonia, hearing loss, short stature) were more common (4). PFIC-2 could rapidly progress to hepatic failure and required liver transplantation. Both PFIC-1 and PFIC-2 have a low to normal level of gamma-glutamyl transferase (γ-GT). In contrast to patients with PFIC-1 or PFIC-2, patients with PFIC-3 usually develop cholestasis in late infancy or adolescence and have an elevated level of γ-GT (4). The signs of cirrhosis (e.g., gastrointestinal bleeding) may appear as the first signs of PFIC-3 in older children or even young adults, with significant variations in clinical symptoms. Diagnosis of PFIC-3 is based on clinical presentation, serum biomarkers, imaging techniques, liver histology and genetic testing (5). Genetic variations in the ATP binding cassette subfamily B member 4 (*ABCB4*) gene are now considered one of the major causes of PFIC-3 (6). To date, more than 500 mutations in *ABCB4* gene have been reported (4), which can be identified through genetic analysis based on next-generation sequencing. Despite this, the clinical manifestations of PFIC-3 patients are very heterogeneous and may be misdiagnosed if without family history. Herein, we report a 16-year-old male mainly manifested with symptoms of pruritus, jaundice, growth retardation, and enlargement of the liver and spleen. The patient was finally diagnosed as PFIC-3 with a compound heterozygote of *ABCB4* gene including a novel heterozygous pathogenic variant. His symptoms were improved dramatically after liver transplantation.

## Case report

A 16-year-old male was hospitalized due to jaundice for 8 days and right lower abdominal pain for 2 days. He had thrombocytopenia, hypoalbuminemia and abnormal clotting. His liver was palpable 3 cm under costal margin, while the spleen edge was palpable 2.5 cm below the left costal margin. He had abnormal results in laboratory examinations: total bilirubin level (TBL) 393.5 µmol/L (normal range 0.0–26.0 µmol/L); direct bilirubin level (DBL) 206.7 µmol/L (normal range 0–6.8 µmol/L); alkaline phosphatase (ALP), 288.9 U/L (normal range 45.0–125.0 U/L); γ-glutamyl transpeptidase activity (GGT), 113.2 U/L (normal range 10.0–60.0 U/L); total bile acid (TBA), 348.4 µmol/L (normal range 0.0–10.0 U/L); serum aspartate aminotransferase (AST), 120.3 U/L (normal range 15.0–40.0 U/L); alanine aminotransferase (ALT), 50.8 U/L (normal range 9.0–50.0 U/L); platelets (PLT), 35 × 10^9^/L (normal range 100–300 × 10^9^/L); serum albumin, 25.5 g/L (normal range 35–51 g/L); prothrombin time (PT), 25.1 s (normal range 12–14 s); activating partial thromboplastin time (APTT), 39.4 s (normal range 31–43 s); and prothrombin activity (PTA), 34% (normal range 75%–100%).Urine porphyrin concentrations were determined and considered within normal limits: hepta-carboxyprophyrin, 1.2 mcg/g creat (normal range 0–2.9 mcg/g creat); uroporphyrin I, 10.8 mcg/g creat (normal range 4.1–22.4 mcg/g creat); and coproporphyrin III, 26.8 mcg/g creat (normal range 4.1–76.4 mcg/g creat). Parameters suggestive of autoimmune or viral hepatitis, such as cytomegalovirus antibody, Epstein-Barr virus, antinuclear antibodies, antimitochondrial antibodies, hepatitis B surface antigen, and hepatitis C virus antibodies, were negative. Serum levels of copper, ceruloplasmin and tumor markers showed no abnormalities. CT documented irregular dilatation of intrahepatic bile ducts, cirrhosis, splenomegaly, collateral veins, ascites and gallbladder wall edema. He was allergic to dust, pollens, fish and seafood with reactions of pruritus. He had a medical history of eczema from around the age of 10 years, treated with topical steroid therapy for several years. Three months ago, he had stopped military training due to itching, tingling and redness on his skin from exposure to the sun. Because of his slow interval growth, he was given a course of growth hormone treatment, which resulted in increased growth velocity. At 16 years, he was 159 cm tall (3rd percentile in the Chinese Centers for Disease Control and Prevention growth charts) and had discontinued growth hormone treatment. There were no risk factors for liver injury, such as drug use, toxins or alcohol abuse. The patient had no similar episodes when he was a child and there were not any factors triggering the clinical onset of disease. His parents were non-consanguineous, and there was no family history of similar symptoms.

**Figure 1:**
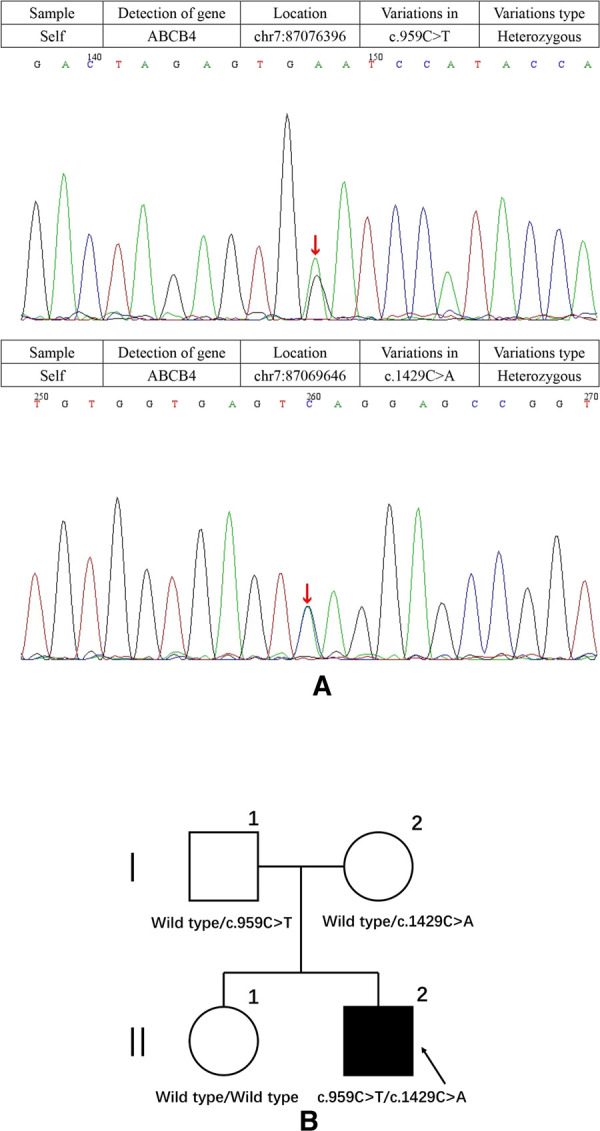
(**A**) results of the *ABCB4* gene test. Gene mutation in c.959C > T and c.1429C > A (red arrows). c.1429C > A was observed due to the exchange of Glu to Lys in codon position 477, which is highly suspected to be a new genetic mutation. (**B**) Pedigrees showing affected proband, his sister and parents.

After hospital admission, the patient was given symptomatic treatment with liver protection, infusion of plasma, albumin, vitamin K1 and anti-inflammatory therapy. A week later, the patient's blood coagulation indexes were corrected, abdominal pain disappeared, and inflammatory markers returned to normal. However, the patient still had severe cholestasis associated with pruritus and jaundice. Accordingly, we adopted a policy of low-dose dexamethasone treatment, and the patient's bilirubin levels had slightly decreased after 3 days but no significant effects after 1 week. Examination of the bone marrow revealed no significant abnormality and primary hematologic dysfunction, or malignancy was excluded. Genetic metabolic diseases were considered, and we performed whole-exome sequencing (WES) and Sanger sequencing with the permission of the patient and his families. Targeted next-generation sequencing, involving 61 genes responsible for genetic disorders of hepatic cholestasis (7) and we detected pathogenic variant in *ABCB4* gene: c.959C > T (p.S320F)/c.1429C > A (p.Q477K) (Figure 1A). c.959C > T (p.S320F) has been reported previously in the literature (8), while c.1429C > A (p.Q477K) is a novel heterozygous pathogenic variant. *ABCB4* c.959C > T (p.S320F) variant was identified in his father and c.1429C > A (p.Q477K) variant was identified in his mother. His parents are heterozygous carriers. Pedigrees for the patient are shown in Figure 1B.

**Figure 2:**
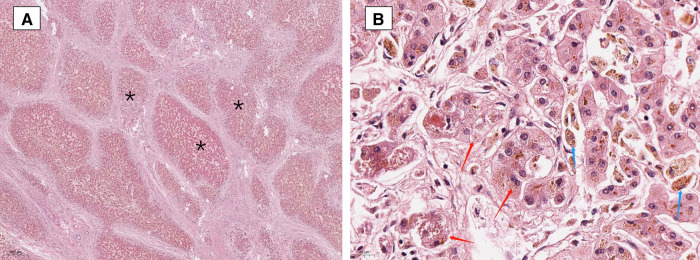
Liver biopsy showing focal nodular hyperplasia (**A** is marked with “*”), prominent bile ductular proliferation within fibrous bands and enlarged hepatocytes with feathery degeneration (red arrows in 2**B**), indicative of intrahepatic cholestasis. Accumulation of pigment in Kupffer cells (blue arrows in **B**). (Hematoxylin and Eosin staining, ×20 and 400).

The patient was treated with ursodeoxycholic acid (UDCA) 15 mg/(kg × d). Sadly, he was insensitive to UDCA and progressed to end-stage liver disease. Multiple liver failure complications plagued him, including jaundice, edema, ascites, spontaneous bacterial peritonitis, splenomegaly, esophageal varices, coagulopathy, and hepatic encephalopathy. At age 17, the patient underwent liver transplantation successfully. The postoperative pathological diagnosis showed intrahepatic cholestasis with progression to cirrhosis (Figure 2). Negative liver tissue immunohistochemistry of BSEP and MDR3 was found in the bile duct membrane. Immunohistochemistry showed absent MDR3 staining, confirming the diagnosis of MDR3 deficiency. The patient was discharged after one month with stable vital signs. Notably, during the 1-year follow-up visit, the patient's liver function was almost in complete remission. Dynamic changes of his liver test are shown in Table 1.

**Table 1:** Dynamic changes of his liver test of the patient.

Examination item	Reference	Admission	7 days after admission	1 day before liver transplantation	30 days after liver transplantation	1 year after liver transplantation	1.5 years after liver transplantation
Alanine aminotransferase (U/L)	9–50	50.8↑	33.6	104.7↑	18.0	10.9	13.2
Aspartate aminotransferase (U/L)	15–40	120.3↑	82.8↑	106.6↑	20.1	16.4	19.1
γ-glutamyltransferase (U/L)	10–60	103.1↑	79.3↑	109.3↑	53.2	35.5	43.5
Alkaline phosphatase (U/L)	45–125	269.7↑	220.1↑	134.3↑	79.2	236.7↑	256.4↑
Total serum bilirubin (umol/L)	5–21	497.2↑	402.8↑	378.6↑	34.3↑	12.3	16.7
Direct bilirubin (umol/L)	<7	244.0↑	199.0↑	186.2↑	11.2↑	2.7	3.5
Albumin (g/L)	36–55	32↓	32.9↓	32.1↓	43.0	41.9	45.4
Total serum bile acid (µmol/L)	<10	353.5↑	442.8↑	317.7↑	29.4↑	6.9	8.2

## Discussion

We present a 17-year male patient, who manifested recurrent episodic jaundice and itching, and eventually progressed to end-stage liver disease. The common causes of liver injury such as viral infections, autoimmune diseases, alcoholic liver disease, and drug-induced liver disease were excluded. Afterward, compound heterozygous mutations in *ABCB4* gene were detected; thus, PFIC-3 was diagnosed.

The patient presented with developmental delay, facial acne, abdominal pain and phototoxicity. Skin symptoms such as cutaneous photosensitivity, increased skin fragility, vesicles, bullae, erosions, crusts and liver damage may occur in patients with porphyria cutanea tarda (PCT) (9). Moreover, Hepatitis C virus antibodies and liver disease in patients with PCT have also been reported (10). Hemophagocytic syndrome was also initially considered, which can be manifested by fever, hepatosplenomegaly, cytopenia, and hemophagocytosis in bone marrow, liver, spleen or lymph nodes (11). However, mutations of the *ABCB4* gene or protein lesions in patients were indicated. The bone marrow examination and urinary porphyrin levels showed no abnormality. Therefore, the diagnosis of PCT or hemophagocytic syndrome was excluded.

Before results from genetic testing were available, the possibilities of other metabolic liver diseases were considered. Dubin-Johnson syndrome can also present with neonatal cholestasis (12), which is caused by genetic variants in *ABCC2* gene (13). Other types of PFIC can't be ignored. PFIC-1 and PFIC-2 were described previously, and the pathogenic genes for type 1–2 PFIC are *ATP8B1* and *ABCB11*, respectively. PFIC-4, PFIC-5 and PFIC-6 are the most recently classified of the PFIC disorders. The tight junction protein-2 (*TJP2*) gene has been linked to PFIC-4, which predominantly presents in childhood (14). PFIC-5 is caused by genetic variants in *NR1H4* gene, which encodes the farnesoid X receptor (FXR). FXR's downstream targets include the genes responsible for PFIC 2 and 3—*ABCB11* encoding the BSEP and *ABCB4* encoding MDR3, respectively (15). PFIC-6 is caused by genetic variants in *MYO5B* gene (16). There are few specific reports of PFIC-6, so further research is needed. Finally, we established the diagnosis of PFIC-3 by genetic analysis.

Genetic analysis is the gold standard for the diagnosis of PFIC-3. The responsible gene has been confirmed to be *ABCB4* gene located on chromosome 7q21, and more than 150 types of diseases are related with the *ABCB4* gene mutations, 50 types of mutations are associated with PFIC3 (17). The *ABCB4* gene codes for the *ABCB4* protein [alias multidrug resistance protein 3 (MDR3)] (18), a *P*-glycoprotein that functions as a phospholipid translocator in canalicular membranes of hepatocytes. It acts as a floppase, transporting lipids from the outer to the inner leaflet of the membrane, and is therefore responsible for phospholipid secretion into the bile (4, 19). Due to phospholipid's role in neutralizing hydrophobic bile salts, *ABCB4* protein defects cause injury to the biliary epithelium and bile canaliculi, ultimately leading to cholestasis (20).

**Figure 3:**
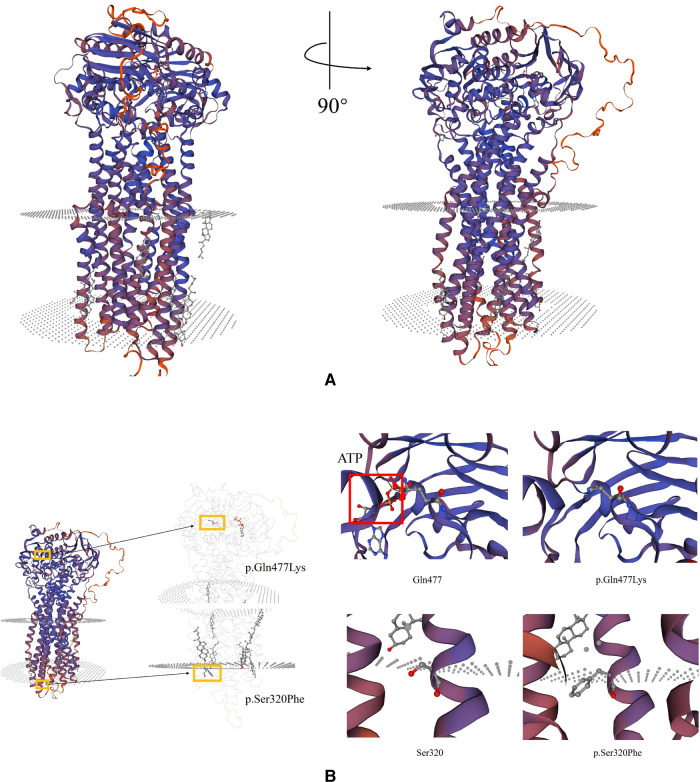
Schematic representation of missense variants p.S320F and p.Q477K on the *ABCB4* protein. (**A**) Predicted tertiary structure of the *ABCB4* novel pathogenic variant by SWISS MODEL. (**B**) Top: Structure modeling of wild type and p.Gln477Lys mutation of *ABCB4*. Top left: The red square shows ATP-bond region. Top right: The mutant shows the loss of ATP-bonds, this might lose the ability to bind and hydrolyze ATP. Bottom: Wild type and p.Ser320Phe mutation of *ABCB4*.

To further explore the deleterious effect of the new mutation, we constructed a three-dimensional model of *ABCB4* protein and this novel pathogenic variant using the SWISS MODEL program (http://swissmodel.expasy.org/) (Figure 3). The molecular effect of the newly identified mutation (c.1429C > A; p.Q477K) was further analyzed by protein modeling using SWISS-MODEL. *ABCB4* protein are composed of two transmembrane domains (TMDs) and two highly conserved nucleotide-binding domains (NBDs). TMDs determine the substrate specificity and the NBDs fuel the transport by binding and hydrolyzing ATP (21). The exchange of Glu to Lys in the highly conserved NBDs (codon position 477) prevents hydrolysis of ATP. The patient's *ABCB4* protein is an ATP hydrolysis deficient mutant.

Heterozygous *ABCB4* variants result in less severe clinical patterns and partially preserve MDR3 protein function. Milder phenotypes of PFIC-3 may present as ICP (22), cholesterol gallstone disease [low phospholipid-associated cholelithiasis (LPAC)] (23), drug-induced cholestasis or liver injury (24), adult idiopathic/cryptogenic cirrhosis (25), transient neonatal cholestasis (26). The patient's parents are heterozygous carriers. They had no similar clinical signs and only had slightly elevated γ-GT, ALT, AST. However, in homozygous or compound-heterozygous variants, PFIC-3 is usually associated with gene defects causing premature truncation or a total failure to produce functional protein (4). Our patient harbors a compound heterozygous mutation of the *ABCB4* gene (c.959C > T/c.1429C > A). The mutation of c.959C > T was common in PFIC-3 patients (27); however, the pathogenicity of c.1429C > A has not been reported so far, and we highly suspected that it is a new genetic mutation, which is speculated to be related to the clinical heterogeneity of the patient. This mutation was not found in the Human Genome Mutation Database (HGMD). An in-silico evaluation of this variation was done with the Mutation tester, Polyphen2, GERP+, and SIFT databases, and all judged this variation as disease causing. The mother of our patient was found to be heterozygous for this mutation (c.1429C > A; p.Q477K). SIFT, PolyPhen2, Mutation Taster, and GERP + were used to predict the protein damage of the mutation, and the results indicated that it was likely to be a pathogenic protein. To the best of our knowledge, this is the first report regarding this novel pathogenic variant of *ABCB4* in a patient with PFIC-3.

Our patient underwent liver transplantation successfully. His laboratory, imaging results as well as clinical symptoms significantly improved. His height went from 159 to 180 cm (97th percentile in the Chinese Centers for Disease Control and Prevention growth charts) within a year. After liver transplantation 1 year later, our patient's liver function was within normal limits except for elevated serum alkaline phosphatase (ALP). Since the patient had no cholestasis or biliary tract injury, we considered that the elevated ALP was due to excessive growth. ALP activity is a diagnostic maker for neo tissue mineralization during bone growth and fracture healing (28). In the process of growth, children's bones grow vigorously. To meet the growth needs, there will be compensatory proliferation of osteoblasts and high activity of serum ALP. The reference range for serum ALP in children aged 4 years is 169–372 U/L (29). The patient showed rapid growth in the last year, so we believe that the increase in ALP is within a reasonable range.

For PFIC-3 patients, the current treatment comprises phenobarbital, UDCA and rifampicin. But our patient had a poor response to UDCA, we predicted the new mutation resulted in loss of expression of the protein MDR3. We found negative liver tissue immunohistochemistry of MDR3 in the explanted liver, which well proved this point. Liver transplantation is the most effective therapy for the patients with homozygous or compound-heterozygous variants.

It was worth noting that immunohistochemistry of BSEP was also negative. Bile salts are secreted into the biliary system through BSEP, which is specifically expressed on hepatocyte canalicular membranes (30). A total of 150 BSEP mutations in *ABCB11* gene have been linked to PFIC-2 so far (31), but no pathogenic variant was tested in *ABCB11* gene. A recent study obtained liver biopsies from PFIC patients and immunostained for BSEP and MDR3 (32). They found a combined BSEP and MDR3 negativity in 35.3% of PFIC-2 group and 66.7% of PFIC-3 group (32). Combined with our patient, we think the absence of both BSEP and MDR3 proteins can exist in PFIC-2 and PFIC-3 patients. Further research is needed for specific mechanism. We should combine with genotyping to help confirm the diagnosis.

In conclusion, for young patients with acute cholestasis, pruritus, jaundice, growth retardation, and enlargement of the liver and spleen, the possibility of inherited metabolic liver diseases should be considered, detailed medical and family history should be collected, and metabolic screening tests as well as gene tests are necessary for correct diagnosis. *ABCB4* mutation diversity contributes to the heterogeneity of clinical manifestations in patients with PFIC-3. This report expands the spectrum of pathogenic mutations of the *ABCB4* gene and provide a basis for rapid and efficient genetic-based diagnosis, genetic counseling for the families of patients, and prenatal diagnosis.

## Data Availability

The original contributions presented in the study are included in the article/Supplementary Materials, further inquiries can be directed to the corresponding author/s.
